# Mental health and quality of life of patients undergoing hematopoietic stem cell transplantation (HSCT) prior to hospitalization: a cross-sectional complete state health study

**DOI:** 10.1080/21642850.2021.1873140

**Published:** 2021-01-28

**Authors:** Maya Corman, Marie-Thérèse Rubio, Aurélie Cabrespine, Isabelle Brindel, Jacques-Olivier Bay, Régis Peffault De La Tour, Michaël Dambrun

**Affiliations:** a Université Clermont Auvergne (UCA), LAPSCO UMR CNRS 6024, Clermont-Ferrand, France; b Service d’hématologie, CHRU Nancy- Hôpitaux de Brabois, Vandoeuvre-les-Nancy, France; c CHU de Clermont-Ferrand, Site Estaing, Service de Thérapie Cellulaire et D’hématologie Clinique Adulte, Clermont-Ferrand, France; d Hôpital Saint-Louis, Service D’hématologie, Greffe de Moelle, Paris, France

**Keywords:** Complete state model of health (CSMH), depression, anxiety, happiness, quality of life, negative and positive psychological dispositions

## Abstract

**Objective:**

The main goal of this cross-sectional study was to examine the relationships between negative/positive psychological dispositions, mental health, and quality of life (QoL) prior to hospitalization among patients undergoing hematopoietic stem cell transplantation (HSCT).

**Method:**

A total of 187 patients (*Mage *= 52.07 years) completed a questionnaire 19.6 days before an allograft. Several positive psychological dispositions (i.e. mindfulness, optimism, and acceptance) and a negative psychological disposition (i.e. experiential avoidance) were assessed. Our dependent variables were mental health (i.e. happiness, depression, and anxiety) and QoL.

**Results:**

In the sample, 56.8% of patients were characterized by an impaired QoL and 56.9% and 21% had, respectively, anxiety and depression levels above the critical threshold (i.e. a score above seven on the Hospital Anxiety and Depression Scale). Anxiety, depression, and happiness were significantly related to the mental component of QoL, whereas physical QoL was only related to depression and happiness. Providing additional support for a complete state health approach, several positive and negative psychological dispositions (i.e. optimism, acceptance, and experiential avoidance) were robustly related to mental illness/wellness and QoL.

**Conclusions:**

These results highlight the importance of improving psychological health and QoL among HSCT patients prior to hospitalization by both promoting positive psychological and health factors and alleviating negative ones.

## Introduction

Hematologic cancers and hematopoietic stem cell transplantation (HSCT) engender major impairments in various dimensions such as quality of life (QoL), chronic physical problems, and psychological distress (Morishita et al., 2013). This is particularly true during HSCT hospitalization and several months and years following transplantation (El-Jawahri et al., 2015; Tecchio et al., 2013). The alteration in QoL between admission to hospital and the 100 days afterwards involves the deleterious effects of HSCT in physical and psychological dimensions (Kisch, Lenhoff, Zdravkovic, & Bolmsjö, 2012). Several studies have investigated the prospective effect of psychological variables, such as depression on recovery from HSCT and survival. They revealed mainly that depressive symptomatology affects survival negatively and positive emotions affect it positively (Hoodin, Uberti, Lynch, Steele, & Ratanatharathorn, 2006; Prieto et al., 2005), while depressive and anxious symptomatologies are predictive of both post-traumatic stress disorder (El-Jawahri et al., 2015) and a poor QoL and well-being in the long term (Amonoo et al., 2019). However, much less is known about the factors that affect both QoL and the mental health of patients prior to hospitalization. To propose efficient interventions before hospitalization and to optimize the social psychological adaptation of patients during a potentially aversive period that requires resilience, identification of protective and deleterious factors related to QoL before bone marrow transplant appears to be particularly relevant.

A systematic review of the literature highlighted an alteration of the overall QoL for people with a hematological cancer diagnosis (Allart-Vorelli, Porro, Baguet, Michel, & Cousson-Gélie, 2015). Additionally, between 13% and 20% of recipients reported anxiety and between 6% and 44% reported depression at different stages of the HSCT process (Artherholt, Hong, Berry, & Fann, 2014). This psychological distress was present even before treatment. For example, patients’ anxiety was significantly higher one week prior to hospitalization than it was at their entrance into the protected area (Prieto et al., 2005) and a third of them reported clinical levels of depression, anxiety, and general distress prior to beginning the protocol (Sherman, Simonton, Latif, Spohn, & Tricot, 2004). In addition, 36% of patients presented a fear of recurrence before the HSCT process, higher than at 100 days and one year after. This fear was positively correlated with depression assessed at the same time (Sarkar et al., 2014). Some studies have also established a significant and negative relationship between emotional distress before and at the early stage of hospitalization and long-term QoL (El-Jawahri et al., 2015) and survival (Hoodin et al., 2006). Thus, the exploration, prior to hospitalization, of the social psychological antecedents of mental health and their relationship with QoL is important.

For this, we adopted the complete state model of health (CSMH) (Keyes, 2005). On the basis of the health definition proposed by the World Health Organization, i.e. ‘a state of complete physical, mental and social well-being and not merely the absence of disease or infirmity,’ the CSMH proposes the investigation of both mental illness and mental wellness variables, which reflect two distinct dimensions with a moderate relationship and have distinct biological correlates. Wellness factors (e.g. optimism and self-acceptance) can buffer the deleterious effects of mental illness and be protective (Keyes, 2005).

Most studies exploring social psychological predictors of mental distress and QoL around HSCT have focused on deleterious factors. Dispositional antecedent factors (e.g. negative affectivity, alexithymia, and neuroticism), transactional variables (e.g. lack of control and high perceived stress), or pre-existing depressive and anxious symptomatologies have been presented in many studies to explain the mental and physical health impairments of patients at different steps of HSCT (Amonoo et al., 2019). Research on protective and beneficial social psychological variables (i.e. mental wellness) is less explored and often treated separately. However, it has been shown that factors such as dispositional optimism, positive affectivity, or resiliency were robustly correlated with both a better QoL and recovery for HSCT patients (Campo, Wu, Austin, Valdimarsdottir, & Rini, 2017; Tomlinson, Yousaf, Vittersø, & Jones, 2017). Recent psychological research has also identified new beneficial factors and processes, such as dispositional mindfulness (Tomlinson et al., 2017), lasting happiness involving inner peace (Dambrun, Ricard, Després, Drelon, & Gibelin, 2012), or acceptance favoring psychological flexibility, which is a core construct of acceptance and commitment therapy (ACT) (Hayes, 2004). Studies of this type of variable are scarcer in psycho-oncology, especially in the case of blood cancers and HSCT. However, dispositional mindfulness already appears as a promising protective factor for patients with a cancer diagnosis (Garland et al., 2016).

Adopting a complete state health approach (Keyes, 2005), we proposed the testing of a relationship model between the psychological dispositions, mental health, and QoL of patients with hematological malignancies prior to their hospitalization to undergo HSCT. The first step of this model concerned the relationship between mental health and QoL. Several studies have indicated that depression and anxiety are robust predictors of patients’ QoL (Smith, Gomm, & Dickens, 2003). Empirical research on the relation between happiness and QoL is not extensive. Lasting happiness involving inner peace is an optimal way of being, which constitutes a psychological resource allowing one to embrace the joys and the pain with which one is confronted (Dambrun & Ricard, 2011). Thus, it could be a protective factor that helps maintain a high QoL in the face of adversity. On this basis, we predicted that anxiety, depression, and lasting happiness would be significantly related to QoL. In a step-by-step regression analysis, we hypothesized that lasting happiness would increase the percentage of explained variance in QoL once mental illness (i.e. anxiety and depression) was statistically controlled for.

The second step of our model was constituted by antecedents of psychological dispositions to mental illness or wellness. Among potential candidates, we selected those that appeared to be the most relevant to the existing literature. For antecedents of mental illness (i.e. negative psychological disposition), we chose experiential avoidance (Larson et al., 2019). Experiential avoidance (i.e. the unwillingness to remain in contact with experiences eliciting unpleasant feelings, thoughts, or bodily sensations), reflects psychological inflexibility, and is known to be a factor maintaining emotional disorders such as anxiety and depression (Spinhoven, Drost, de Rooij, van Hemert, & Penninx, 2014). Optimism (Kenzik, Huang, Rizzo, Shenkman, & Wingard, 2015), dispositional mindfulness (Garland et al., 2016), and acceptance (Secinti, Tometich, Johns, & Mosher, 2019), positive psychological factors now well studied in psycho-oncology, were included as discrete antecedents of mental wellness (i.e. positive psychological dispositions). Amonoo et al. (2019) suggested that some positive psychological resources such as optimism are related to better mental and physical QoL and health among patients benefiting from HSCT. Larson et al’s study (2019) is the only research in the case of HSCT to have explored the effect of dispositional mindfulness and its components (i.e. observing, describing, non-reacting, non-judgmental, and acting with awareness). Their results reveal a prospective effect of dispositional mindfulness and the facet of non-judgment, a psychological process close to acceptance and that we can oppose to experiential avoidance, on anxiety and depression symptomatology following HSCT. Acceptance is often studied as a coping strategy and barely as a psychological disposition despite its role in reducing cancer-specific distress (Secinti et al., 2019). Consequently, acceptance and dispositional mindfulness can be suitable to explore new psychological resources involved in positive and negative outcomes of HSCT. Using multiple regression analyses, we tested the robustness of the relationship between each negative/positive psychological disposition and mental illness/wellness (i.e. anxiety and depression or happiness), and QoL.

## Method

### Participants

The study protocol was presented to 275 patients affected by a hematological disease and candidates for an allogeneic hematopoietic stem cell transplantation (HSCT). Of these, 187 (*M*_age _= 52.07, *SD *= 13.22, ranging from 19 to 72 years old) from the three hospital centers of Paris, Nancy, and Clermont-Ferrand in France took part.

We estimated the required sample size for sufficient correlation power (90%). On the basis of the correlations between optimism and QoL in the context of HSCT reported by Kenzik et al. (2015); (i.e. *r* = 0.39 between optimism and physical QoL) the minimum required sample size was 65. The ethical committee Sud-Est III (IRB 2017-026 B) approved the study. Informed written consent was obtained from each participant.

### Procedure

All participants were informed of the study during the pre-graft interview, generally provided between 6 and 8 weeks before hospitalization, and they read an information note. They were given 15 days to decide whether they would participate or not. Then, they filled out an informed consent form and completed a self-report questionnaire assessing several psychological dimensions and socio-demographic variables with an average of 19.6 days (*SD *= 14.14; Range = [0; 64]) before their transplantation. For two of them, it was impossible to complete the questionnaire before their hospitalization, despite their agreement, so they did so at the transplantation time in hospital.

### Measure

*Quality of life*. We used the SF-12 (Gandek et al., 1998), which is a short version of the SF-36 that assesses mental and social (*α* = .70) and physical QoL (*α*_t_ = .71) in 12 items subdivided into 8 categories (i.e. physical activity, life and relationships with others, physical pain, perceived health, vitality, limitations due to mental state, limitations due to physical state, mental health). Each item score was converted into standardized data before computation of a global score for physical QoL and mental and social QoL. A greater score on each of the subscale indicates a higher level of QoL.

*Mental health*. We assessed anxiety, depression, and happiness. Anxiety and depression symptomatologies (during the last week) were measured with the Hospital Anxiety and Depression Scale (HADS) (Zigmond and Snaith, 1983). Seven items estimate anxiety symptomatology (*α* = .76) (e.g. I feel tense or ‘wound up’) and seven items assess symptoms of depression (*α* = .70) (e.g. I’m enjoying the same things I used to enjoy). Participants rated items on a scale from 0 to 3 and the total score was computed from the sum of seven anxiety items (range from 0 to 21) and seven depression items (range from 0 to 21). Greater values on each subscale indicate greater anxiety and/or depression. Happiness (in the course of life) was assessed with the Subjective Authentic − Durable Happiness Scale (SA − DHS) (Dambrun et al., 2012) (*α* = .95) including 13 items assessing regular levels of happiness or inner peace, for example. Score was totaled from the 13 items. This is a seven-point Likert scale (from 1 ‘very weak’ to 7 ‘very high’). Higher scores on this scale represent higher level of happiness.

*Negative psychological disposition*. Experiential avoidance was measured with the Avoidance and Fusion Questionnaire for adults (AFQ) (Fergus et al., 2012) (*α* = .88). This four-point Likert scale (from 0 ‘not true at all’ to 4 ‘very true’) comprises items measuring the degree of adherence with 17 statements (e.g. I can’t stand feeling pain or hurt in my body). The sum of all 17 items was computed to obtain a total score (range from 0 to 68). More the score on this scale is high more the level of experiential avoidance is high.

*Positive psychological dispositions*. Three constructs were used to measure positive psychological dispositions: optimism was measured using the Life Orientation Test Revised (LOT-R) (Scheier, Carver, & Bridges, 1994) (*α* = .75) in 10 items (e.g. In uncertain times, I usually expect the best), comprising four fillers. Participants rated items on a scale from 1 (Disagree at all) to 7 (Totally agree). The score of optimism was totaled with 6 items (comprising 3 reversed items) revealing that more the score is high, more the participant shows dispositional optimism. The Five Facets Mindfulness Questionnaire (FFMQ) (Baer et al., 2008) was used to measure dispositional mindfulness. The FFMQ assesses in 39 items five facets of dispositional mindfulness (i.e. observation, description, aware actions, non-judgment of inner experience, non-reactivity). This measure is a five-point Likert scale (from 1 ‘ never or very rarely true’ to 5 ‘very often or always true’). The total score of dispositional mindfulness was obtained by summing all the 39 items. Greater values on this scale describe greater level of dispositional mindfulness. The reliability was adequate (*α* = .87). Finally, acceptance was assessed with the Acceptance and Action Questionnaire II (AAQ II) (Bond et al., 2011) (*α* = .81). This seven-point Likert scale (from 1 ‘never true’ to 7 ‘always true’) measures acceptance with a totaled score computed from 10 items (e.g. My thoughts and feelings do not get in the way of how I want to live my life). More the total score is high more participant experiences high level of acceptance.

*Controlled variables*. The controlled variables were socio-demographic (i.e. age, sex, marital status, and educational level) and the medical variables were regular (in the daily life) alcohol consumption, smoking and physical activity (yes/no binary questions), body mass index (computed from weight and height provided by the participant), sleeping hours, and type of disease. Except the type of disease, all these information were self-reported. The number of days between the completion of questionnaire and transplantation has been controlled too.

### Data analysis

First, we investigated the relationships between mental illness, mental wellness, and the two components of the QoL scale (mental and physical; SF-12) by means of Pearson correlation analyses. Then, we tested the hypothesis that lasting happiness would increase the percentage of explained variance in QoL once mental illness (i.e. anxiety and depression) was statistically controlled for in a step-by-step regression analysis. Second, we investigated the relationships between psychological dispositions and mental health by means of Pearson correlation analyses. Due to the number of correlations (*N* = 30), we computed the adjusted *p* values (i.e. q values; q = p × N/rank). Finally, the robustness of the relationships between psychological dispositions and mental health was tested in a series of multiple regression analyses. Variance inflation factors (VIFs) were calculated to check for multicollinearity.

## Results

### Descriptive statistics

The sample included 41.9% of women. In total, 65.7% were married or in a relationship, 22.1% were single, 10.5% were divorced and 1.7% were widowed. Overall, 46.3% of our sample had an educational level beyond the license degree and 69.6% were employed. Seventeen percent had myelodysplastic syndrome, 10.4% had myeloproliferative neoplasia, 12.1% had a non-Hodgkin Lymphoma, and 35.7% were candidates for an allograft for acute leukemia. The remaining 25.1% of the population was affected by other hematological disease such as precursors lymphoid neoplasm, chronic leukemia, multiple myeloma or Hodgkin lymphoma. Ninety-four percent were having their first transplant. Table 1 depicts characteristics of the sample.

**Table 1.** Descriptive statistics for socio demographic and medical variables (*N* = 187).

	% (excluding missing values)	Mean (SD)
S**ocio** d**emographic** v**ariables Age**		52.07 (13.22)
Sex *(women)*	41.9	
Marital status		
*Single*	22.1	
*In a relationship*	19.3	
*Married*	46.4	
*Widowed*	1.7	
*Divorced*	10.5	
Educational Level *(post-graduate)*	46.3	
Socioprofessional Category		
*Employed*	69.6	
*Others (retired, without professional activity, student, ...)*	30.4	
M**edical variables**		
Disease status Acute leukemia Myelodysplastic syndrome Myeloproliferative neoplasia Non Hodgkin lymphoma Others (Hodgkin lymphoma, chronic leukemia, …)		
*Acute Leukemia*	35.7	
*Myelodysplastic syndrome*	17	
*Myeloproliferative neoplasia*	10.4	
*Non Hodgkin lymphoma*	12.1	
*Others (Hodgkin lymphoma, chronic leukemia, ...)*	25.1	
Alcohol consumption *(yes)*	30.8	
Smoking *(yes)*	15.8	
Physical activity *(yes)*	45.3	
Body mass index		24.92 (4.61)
Sleeping hours		7.42 (1.15)
Numbers of transplantation		
*One*	94	
*>1*	6	

### Mental health and quality of life

A series of one-sample *t*-tests revealed that the sample scored lower on both the mental and physical components of the QoL scale before hospitalization (*M *= 45.7 and *SD *= 10.54, and *M *= 38.36 and *SD =* 10.12, respectively) than a sample of 2743 from a French population (*M *= 48.4, *SD *= 9.4, *p*s* *<* *.001; *M *= 51.2, *SD *= 7.4, respectively; *p*s* *<* *.001) (Gandek et al., 1998). A significant proportion of women and men present scores above the normal on both anxiety and depression subscales (i.e. a score above seven on the HADS). Finally, women had a significantly lower level of lasting happiness than men (*t *= −2.31, *p*s* *<* *.03) (Table 2).

**Table 2.** Means, standard deviations (*SD*) and minimum (min)/maximum (max) for quality of life, mental and physical health components, and positive/negative psychological dispositions.

	Mean (*SD*)	Min	Max	*N*	Mean women (SD)	Mean men (SD)
*Quality of life components*						
Mental & Social QoL (SF-12)	45.69 (*10.54)*	21.35	66.04	183	45.54 (10.63)	45.82 (10.51)
Physical QoL (SF-12)	38.36 (*10.12*)	16.44	62.54	183	37.60 (10.46)	38.99 (9.83)
*Mental health components*						
Anxiety (HAD-A)	8.53 (*3.69*)	0	19	181	9.09 (3.65)	8.07 (3.68)
Score > 7 (%)					62.7	52.5
Depression (HAD-D)	5.22 (*3.32*)	0	19	181	4.98 (3.09) 17.1	5.42 (3.52) 24.2
Score > 7 (%)					17.1	24.2
Happiness (SA-DHS)	60.57 (*14.38*)	17	91	183	57.85 (13.33)	62.74 (14.88)
*Positive psychological dispositions*						
Mindfulness (FFMQ)	131.91 (*16.27*)	74	177	186	132.87 (17.47)	131.15 (15.29)
Optimism (LOT-R)	29.65 (*6.41*)	11	42	185	29.15 (6.21)	30.06 (6.58)
Acceptance (AAQ)	47.05 (*9.32*)	14	67	184	46.35 (9.19)	47.61 (9.43)
*Negative psychological disposition*						
Experiential Avoidance (AFQ)	32.90 (*12.08*)	4	63	184	34.62 (11.74)	31.51 (12.23)

Note*:* FFMQ: Five Facets Mindfulness Questionnaire; SA-DHS: Subjective Authentic-Durable Happiness scale; LOT-R: Life Orientation Test-Revised; AAQ: Acceptance and Action Questionnaire; AFQ: Avoidance and Fusion Questionnaire; HAD-A: Hospital Anxiety and Depression-Anxiety; HAD-D: Hospital Anxiety and Depression-Depression; SF-12: Short Form-12; SD: Standard Deviation.

Psychological dispositions, mental health, and quality of life were not significantly affected by the type of disease (ie.g. acute leukemia, myelodysplastic syndrome, and so on), the other medical variables (i.e. alcohol consumption, smoking, and sleeping hours) and the time between the completion of questionnaire and transplantation: all *p*s* *>* *.10. As a result, we decided to analyze the data for all patients regardless of their disease and the other medical variables.

Table 3 presents the Pearson correlations between anxiety, depression, happiness, and QoL. First, anxiety and depression were significantly correlated. Happiness was negatively related to both anxiety and depression. Second, and as expected, anxiety, depression, and happiness were significantly related to the mental component of QoL. Only depression and happiness correlated significantly with the physical component of QoL. Adjusting for significant covariates revealed the same basic findings (i.e. Body Mass Index for anxiety, age and physical activity for depression, and sex and marital status for happiness). As predicted, in a step-by-step regression analysis, happiness significantly increased the percentage of explained variance in mental QoL once mental illness (i.e. anxiety and depression) was statistically controlled for (*R*^2^ change = 0.025, *p* < .009). This was not the case for the physical component of QoL, where happiness only marginally increased the percentage of explained variance once depression was statistically controlled for (*R*^2^ change = 0.016, *p* < .078).

**Table 3.** Correlations (95% CI) between various dimensions of negative/positive psychological dispositions, mental health (anxiety, depression, and happiness) and quality of life.

	*Mental health*			*Quality of life (QoL)*	
	Anxiety	Depression	Happiness	Mental SF-12	Physical SF-12
*Mental health variables*					
Anxiety	–				
Depression	.48*** (0.35, 0.58)	–			
Happiness	−.48*** (−0.59, −0.36)	−.38*** (−0.50, −0.25)	–		
*Quality of life (QoL)*					
Mental SF-12	−.45*** (−0.56, −0.33)	−.54*** (−0.64, −0.43)	.42** (0.29, 0.53)	–	
Physical SF-12	−.02 (−0.16, 0.13)	−.29*** (−0.42, −0.15)	.16* (0.01, 0.30)	.12 (−0.02, −0.26)	–
*Negative psychological disposition*					
Experiential avoidance	.57*** (0.46, 0.66)	.37*** (0.23, 0.49)	−.37*** (−0.49, −0.23)	−.45*** (−0.56, −0.32)	−.12 (−0.26, 0.03)
*Positive psychological dispositions*					
Optimism	−.53*** (−0.63, −0.41)	−.44*** (−0.55, −0.32)	.55*** (0.43, 0.64)	.38*** (0.25, 0.50)	.05 (−0.10, 0.19)
Mindfulness	−.45*** (−0.56, −0.32)	−.31*** (−0.44, −0.18)	.38*** (0.25, 0.50)	.33*** (0.19, 0.45)	.03 (−0.12, 0.17)
Acceptance	−.58*** (−0.67, −0.48)	−.44*** (−0.55, −0.31)	.51*** (0.40, 0.61)	.43*** (0.30, 0.54)	.05 (−0.09, 0.20)

Note: ***q < .001; **q < .01; *q < .05 (*q* = *p* × N/rank).

### Negative/positive psychological dispositions, mental health, and quality of life

Table 3 presents the correlations between the various dimensions of negative/positive psychological dispositions, mental health, and QoL. While physical QoL was not significantly related to these various dimensions, all the assessed psychological dispositions were significantly correlated – in the expected direction – with anxiety, depression, happiness, and mental QoL (all ps < .001). After adjusting for significant socio-demographic and medical covariates of anxiety, depression, happiness, and mental QoL (see above), we found similar results.

We went further by examining the robustness of these relationships in a series of multiple regression analyses with experiential avoidance, optimism, mindfulness, and acceptance as simultaneous independent variables. Variance inflation factors (VIFs), which were ranged from 1.45 to 2.22, indicated an absence of multicollinearity. As shown in Table 4, experiential avoidance, optimism, and acceptance were robustly related to anxiety. While only optimism was robustly related to depression, only experiential avoidance was significantly related to mental QoL. Finally, both optimism and acceptance were robustly related to happiness.

**Table 4.** Standardized beta coefficients in multiple regression analyses of the relationships between negative/positive psychological dispositions, mental health (anxiety, depression, and happiness) and mental quality of life.

	Standardized beta	Unstandardized beta	95% CI	*p*-value	*R*-squared
*DV: Anxiety*					
Experiential avoidance	0.29***	0.22	0.11, 0.32	0.001	
Optimism	−0.22**	−0.11	−0.18, −0.04	0.003	
Mindfulness	−0.12+	−0.15	−0.31, 0.02	0.085	
Acceptance	−0.21*	−0.12	−0.22, −0.03	0.012	
					0.455
*DV: Depression*					
Experiential avoidance	0.12	0.08	−0.03, 0.20	0.15	
Optimism	−0.26**	−0.12	−0.19, −0.04	0.02	
Mindfulness	−0.06	−0.07	−0.24, 0.11	0.46	
Acceptance	−0.17+	−0.09	−0.19, 0.01	0.08	
					0.256
*DV: Happiness*					
Experiential avoidance	−0.03	−0.05	−0.29, 0.20	0.71	
Optimism	0.35***	0.36	0.20, 0.52	0.001	
Mindfulness	0.09	0.23	−0.15, 0.61	0.23	
Acceptance	0.24**	0.28	0.07, 0.49	0.009	
					0.356
*DV: Mental QoL*					
Experiential avoidance	−0.27**	−4.02	−6.48, −1.56	0.002	
Optimism	0.15+	1.44	−0.20, 3.07	0.085	
Mindfulness	0.08	2.00	−1.92, 5.90	0.31	
Acceptance	0.13	1.50	−0.68, 3.68	0.18	
					0.261

Note: ***p < .001; **p < .01: *p < .05; +*p* < .10.

## Discussion

Under a complete state health approach, the main objective of this study was to explore, prior to hospitalization for an allogenic HSCT (which engenders emotional distress and impairment of QoL), the relationships between negative/positive psychological dispositions, mental health (i.e. depression, anxiety, and happiness), and QoL. We predicted that anxiety, depression, and happiness would be significantly related to QoL. We also investigated the role of each positive/negative psychological disposition as potential correlates of mental wellness/illness (happiness, anxiety, and depression), and QoL. Prior to hospitalization and transplantation, it appeared that anxiety, depression, and happiness were significantly related to mental QoL, whereas only depression and happiness were significantly related to physical QoL. This confirms previous studies, which revealed that depression represents a major issue in the context of HSCT (El-Jawahri, Vandusen et al., 2015; El-Jawahri, Traeger et al., 2015). The present study complements previous studies by focusing on the relationship between depression and QoL *before hospitalization and transplantation*. The mental health of our sample just before hospitalization and transplantation indicated psychological distress even before their entrance into the protective area (i.e. a germ-free room to protect patient against infection during hospitalization period). Indeed, more than 56% of patients (62.6% of women and 52.5% of men) suffered anxiety symptoms. Despite a decline in anxiety symptomatology over the time course of hospitalization (Kisch et al., 2012; Tecchio et al., 2013), anxiety can be a sign of a maladaptive adjustment to the process, which should be taken account in the psychological care before transplantation. The same observation is valuable for depression. We can observe that men present a significant higher depressive symptomatology than women. This result is not consistent with a previous finding showing that women report more depressive and anxiety symptomatology before their transplantation (Morishita et al., 2013). Results depicting differences in the lower level of happiness, a component of subjective well-being for women compared to men, can be in part explained by age (i.e. older women report lower levels of subjective well-being than men and most of our sample is older than 50 years old), poorer social and health conditions of women over time (Pinquart & Sörensen, 2001).

The fact that anxiety and depression were significantly related to QoL before hospitalization and transplantation highlights the importance of detecting and engaging in preventive interventions before hospitalization to buffer impairments of QoL. In this respect, several additional results invited us to consider positive interventions as potentially relevant to reduce the depressive and anxious symptomatology before HSCT. Firstly, controlling for depression, happiness added a significant percentage of explained variance in mental QoL prior to hospitalization and transplantation. It is not surprising that happiness affects or has been affected by QoL according to the *Life Quality and Well-being* model which demonstrates a close relationship between subjective well-being (including happiness) and QoL (Skevington & Böhnke, 2018) and other studies in psycho-oncology (Tessier, Blanchin, & Sébille, 2017). However, this relationship is underexplored in the HSCT case. Secondly, both positive and negative psychological dispositions were found to be robustly related to mental health. Thus, targeting happiness, depression, anxiety, and the variables that improve them in an intervention adapted to the circumstances and context of allograft before hospitalization and transplantation represents a potentially fruitful avenue. Some results provided further direction. Most of the psychological dispositions we investigated were robustly related to anxiety, depression, happiness, and mental QoL prior to hospitalization and transplantation. Adjusting for other psychological dispositions, only mindfulness was not robustly related to our various dependent variables. Research has already demonstrated that mindfulness fosters positive emotions and optimism. Thus, its effect on health would be accounted by these variables (Garland et al., 2016; Tomlinson et al., 2017). In the present research, the relationship between trait mindfulness and mental health vanished when optimism was statistically controlled for. This is consistent with the mindfulness-to-meaning theory, which proposes that mindfulness improves mental health in the face of cancer through a chain of positive psychological processes (Garland et al., 2016). In addition, acceptance and experiential avoidance emerged as robust predictors of several mental health variables. Acceptance and experiential avoidance are two extremes of a same continuum contributing to, respectively, mental health and illness. In psycho-oncology, acceptance is barely explored as a disposition (Secinti et al., 2019), as is avoidance (Aguirre-Camacho et al., 2017), which is more studied as a coping strategy. However, growing empirical and interventional research in this field suggest that acceptance and experiential avoidance contribute to the reduction in, or maintenance of, psychological distress (i.e. anxiety, depression and post-traumatic stress disorder) and affect positively or negatively the QoL (Aguirre-Camacho et al., 2017). Thus, targeting some of these variables using tools from the third wave of cognitive and behavior therapy, such as mindfulness-based stress reduction (MBSR) for mindfulness (Grossman, Niemann, Schmidt, & Walach, 2004) or ACT for acceptance and experiential avoidance (Hulbert-Williams, Storey, & Wilson, 2015), should be beneficial for patients prior to their hospitalization and transplantation, and also for their post-HSCT lives (Larson et al., 2019). In the present study, illness prior to hospitalization (i.e. depression) was mainly associated with a positive psychological disposition (i.e. optimism). This is consistent with the CSMH, which proposes that mental and physical health is not merely the absence of disease (i.e. languishing) but also a state of flourishing (i.e. life satisfaction, personal growth, and so on), and that positive and negative dimensions of health are strongly intertwined (Keyes, 2005).

### Study limitations

This study has some limitations. Firstly, the correlational nature of this study meant that causality between variables could not be addressed. A longitudinal design would allow a more precise identification of the prospective effects of psychological dispositions (e.g. through bio-behavioral pathways) on mental health (Knight, Lyness, Sahler, Liesveld, & Moynihan, 2013). The period during which the questionnaire was filled out could have affected our results. Indeed, some of the patients completed it several weeks before transplantation, whereas others completed it a few days before the allograft. We know that quality of life and psychological distress can quickly fluctuate over time and according to the different stages of the process (Kisch et al., 2012; Tecchio et al., 2013). We could not prevent this given the heterogeneity of the transplant process through the three medical centers but this should be taken into account more directly in future studies. Finally, the participants were quite well-educated and mainly of French nationality, and the majority were married. Therefore, the findings cannot be generalized.

### Clinical implications

This study highlights the importance of addressing both positive and negative dimensions of health in the potentially aversive context of HSCT, and the need to develop and engage in prevention intervention given the high psychological distress levels of patients before hospitalization and transplantation. More studies in psycho-oncology and, moreover, in the case of HSCT, are needed to better explore the different pathways through which these positive psychological dispositions interact with mental health (e.g. happiness, overall well-being) and illness (e.g. depression, anxiety). These investigations would allow building, for example, a model based on CSMH or Life Quality and Well-being applied to HSCT cases. Such research is necessary before proposing a psychological intervention. However, these first results and the growing evidence suggesting that positive psychological resources such as acceptance help patients to face illness and treatment such as HSCT, provide some robust elements warranting the use of positive and third-wave-based interventions with patients undergoing HSCT.

Even if positive and third-wave-based interventions may promote the emergence of psychological resources to cope with HSCT, according to the growing literature about positive interventions in cancer (Casellas-Grau, Font, & Vives, 2013), HSCT remains a particular treatment raising different issues and hindrances, preventing a standard implementation of interventions, especially before hospitalization and transplantation. So, identifying specific factors in order to target them through intervention is essential to benefit patients. This study have provided some elements in this direction to improve patient care pathway.
